# Systematic review of mRNA expression in human oocytes: understanding the molecular mechanisms underlying oocyte competence

**DOI:** 10.1007/s10815-023-02906-9

**Published:** 2023-08-10

**Authors:** Xavier Viñals Gonzalez, Arwa Almutlaq, Sioban Sen Gupta

**Affiliations:** Institute for Women’s Health, Preimplantation Genetics Group, University College London, 84-86 Chenies Mews, Bloomsbury, London, WC1E 6HU UK

**Keywords:** Human oocyte, Quality, Pathway, mRNA

## Abstract

The biggest cell in the human body, the oocyte, encloses almost the complete machinery to start life. Despite all the research performed to date, defining oocyte quality is still a major goal of reproductive science. It is the consensus that mature oocytes are transcriptionally silent although, during their growth, the cell goes through stages of active transcription and translation, which will endow the oocyte with the competence to undergo nuclear maturation, and the oocyte and embryo to initiate timely translation before the embryonic genome is fully activated (cytoplasmic maturation). A systematic search was conducted across three electronic databases and the literature was critically appraised using the KMET score system. The aim was to identify quantitative differences in transcriptome of human oocytes that may link to patient demographics that could affect oocyte competence. Data was analysed following the principles of thematic analysis. Differences in the transcriptome were identified with respect to age or pathological conditions and affected chromosome mis segregation, perturbations of the nuclear envelope, premature maturation, and alterations in metabolic pathways—amongst others—in human oocytes.

## Introduction

Chromosome aberrations which occur along nuclear maturation during oogenesis are the major, but not single, determinants of poor oocyte quality. During its genesis and differentiation process, the female oocyte coordinates appropriate chromosome segregation and ooplasm and molecular organisation (cytoplasmic maturation). Although the latter process is poorly understood, it is known to involve a physical growth of the oocyte, provided by the active synthesis of macromolecules, proteins and stage-specific and timely messenger RNAs (mRNA) and their specific posttranslational modification [1]. mRNAs work as templates of genes which move from the nucleus of the cell to the cytoplasm, where they will be translated to a series of amino acids to subsequently form proteins. Therefore, protein expression is linked to mRNA quantity, which is conditioned by the equilibrium of transcription rates (the process of making RNA copies from DNA gene sequences) and decay. However, studies have shown that it is not only quantity but posttranscriptional modifications of mRNAs such as capping, splicing and polyadenylation that govern mRNA storage, recruitment, translation and decay during oocyte maturation and early embryogenesis [2–4]. A global shift in maternal mRNA translation, which is crucial for meiotic progression, fertilization, and embryo development, has been reported to coincide with the oocyte’s re-entry into the meiotic cell cycle [5]. This switch involves the repression of mRNAs that are highly active in quiescent oocytes and the activation of mRNAs that were repressed. In the recent years, the use of single-cell RNA sequencing (scRNA-seq) technology has contributed to investigating transcriptional differences in oocytes [6]. Through the present systematic review, authors aim to identify quantitative differences in transcriptome of human oocytes that may link to patient demographics that could affect oocyte competence.

## Methods

### Design and search strategy

A systematic review was performed to allow the identification, screening, and summary of published research findings in a structured and reproducible method. Such analysis was conducted across three electronic databases (MEDLINE, EMBASE and Google Scholar). A manual search of original articles was also performed to allow integration of further studies. The latest search was carried out on 20/03/2023. The search terms used were as follows: messenger RNA OR mRNA AND profiling OR gene expression OR transcripts AND quantitative OR research article AND oocyte OR egg AND quality OR competence OR development OR maturation OR fertilisation OR embryo OR outcome AND human.

The study selection process followed the PRISMA (preferred Reporting Items for Systematic Reviews and Meta-Analysis) guidance [7]. Search results from the three databases were compared and duplicates were removed. Furthermore, results were screened by title and abstract to establish relevance for the present review. The search was also performed by a second person to corroborate the results.

Inclusion criteria for studies were as follows:

- Original research articles, publication in peer-reviewed journals.
- Recent publication date (2015-2023).
- Include the study of human oocytes and their mRNA content.

Exclusion criteria for studies were as follows:

- Article not accessible in English.
- Abstracts, reviews, letters and editorials.
- Non-peer reviewed articles.

### Quality assessment

KMET score was used to critically appraise eligible articles as described by Kmet et al., 2004 [8]. This allows the assessment of quantitative research based on 14 criteria, each of them is scored as “not met/not applicable” (0 points), “partially met” (1 point) or “met” (2 points). The total score is converted to a percentage (over a maximum of 28 points). As described by Kmet et al. (2004) a low cut-off point of 55% was used as an inclusion/exclusion parameter.

### Data extraction and synthesis

Table 1 summarises the study details, including purpose, methodology, genes studied, cohort description, main findings and quality assessment score. Quantitative data derived from the included studies was analysed following a thematic analysis approach [24], allowing the identification across different study designs to be combined and understood.

**Table 1:** Summary of the selected studies. Purpose, methodology, content, and findings are described for each of the studies, together with their corresponding KMET score

Reference	Purpose Of Study	Methodology	Genes	Cohort	Findings	Limitations	KMET
Novin et al., 2015 [9]	Transcript expression levels in various stages of oocyte maturation	qRT-PCR Single Cell	*MT-COI1, NRF1, TFAM*	27 oocytes (8 MII, 9 MI, 10 GV), 9 patients	The mean expression of mitochondrial encoded gene (*MT-COI1*) and two nuclear encoded genes (*NRF1 and TFAM*) was relatively low in GV-stage oocytes and varied in expression in MI and MII stage oocytes.	(1) Sample size. (2) Only 20-35 years old and apparently healthy patients included	61
Hoseini et al., 2016 [10]	To evaluate the effect of transferring the cytoplasm of a mature to a GV oocyte on nucleus and cytoplasmic maturity of the GV oocyte at the mRNA level	qRT-PCR Pooled oocytes	*GDF9, BMP15, ATPase6, AURKC, CDC25, CDC20, MAD2L1, BUB1, ATR, ATM, TP53, NAIP, BRCA1*	120 GV (40 intact, 40 IVM and 40 cytoplasmic transfer	54% decrease in ATPase6 in the cytoplasmic transfer group to others. Genes involved in meiosis increase in the in vitro matured oocytes but not in the intervention group. Cytoplasmic transfer disrupts the expression of genes involved in cytoplasmic maturity.	(1) Pooling may mask some individual differences in oocytes in the different groups.	78
Liu et al., 2016 [11]	mRNA expression profile of ECAT1 in human tissue oocytes and pre-implantation embryos	qPCR Single cell and embryos	*ECAT1*	260 GV stage oocytes, adult ovarian tissue and poor Day 3 cleavage embryos	ECAT1 mRNA declined from oocyte to zygote and then began to increase until the eight cell embryo stage, to then decline in embryos at the morula stage; and mRNA was barely detectable at the blastocyst stage.	(1) Sample size for ovarian tissue comparisons.	68
Li et al., 2016 [12]	To determine whether placenta-specific 8 (PLAC8) is expressed in human oocytes and embryos	qPCR Single cell and embryos	*PLAC8*	3 MII oocytes, 9 cleavage embryos and 3 blastocyst for mRNA analysis	PLAC8 mRNA expression was undetectable in MII oocytes, two to eight cell embryos. PLAC8 mRNA expression was detected in one morula and three blastocysts. PLAC8 protein was detected in oocytes and embryos at every stage using immunofluorescence.	(1) Limited sample size. (2) In vitro model cannot fully mimic the normal human endometrium in vivo.	60
Barragan et al., 2017 [13]	Oocyte transcriptome changes associated with female age and ovarian reserve.	Microarray and qPCR Single cell	Sequencing (all)	36 vitrified/warmed MII stages from 30 women	Age and ovarian reserve classification resulted in 17 and 22 differentially expressed mRNA, respectively. *ANXA5 and DUXAP10* were increased in oocytes in older women with low antral follicle count. *SPCS2 and PRRG1* were increased in oocytes in older women with low AFC. >70% differential expressed genes were ncRNAs.	(1) No function has yet been established for some differentially expressed genes. (2) Sample size.	82
Canosa et al., 2017 [14]	mRNA expression of zona pellucida genes in oocytes and CCs	qRT-PCR Single cell	*ZP1, ZP2, ZP3, ZP4, ENFB2*	98 oocytes (80 MII, 10 MI, 8 GV) and 98 corresponding CCs	In the oocytes, all ZP mRNA were detectable and the expression level in mature versus immature showed a significant decrease in ZP1, 2 and 4. ZP3 expression showed a non-significant decrease. ZP mRNA expression is related to oocyte maturity and fertilisation capacity.	(1) Patients with different infertility aetiology. (2) Large range of age [22-42] considering sample size.	64
Ruebel et al., 2017 [15]	To compare oocyte gene expression profiles and follicular fluid content from overweight versus normal weight women	SMART Ultra Low Input RNA kit and qRT-PCR Single cell	Sequencing (all)	39 oocytes (10 MII, 18, MI, 11 GV) from 24 women. Corresponding FF was analysed	Overweight women had an increased FF triglyceride, leptin, and c-reactive protein levels. Changes in oocyte transcriptome at different maturation stages of overweight women compared with those with those of normal weight women. *CXCL2* and *DUSP1* were upregulated while *TWIST1, ID3, GAS7 and TXNIP* were downregulated, in obese women.	(1) Documented infertility of participants and ovarian stimulation may induce changes in mRNA profiles. Sample size.	78
Zhang et al., 2018 [16]	To analyse the gene expression dynamics throughout folliculogenesis by exploring the transcriptomes of the human oocyte and granulosa cells, at five key stages of follicular development in vivo	RNA-Seq Single cell	Sequencing (all)	80 oocytes and 71 granulosa cell	*NTF-4* and *LCP2* preferentially expressed in oocytes of antral stages. NOTCH signalling pathway in granulosa cells is activated via oocyte driven mechanisms.	(1) Limited range in female age (24-32y)	92
Ferrero et al., 2019 [17]	Do oocytes from women with ovarian endometriosis have a different transcriptomic profile than those from healthy women?	SMART-Seq V4 Ultra-low input RNA sequencing kit Single Cell	Sequencing (all)	16 MII from 7 endometriosis patients; 16 MII oocytes from 5 healthy patients	Amongst the top 20 significantly differentially expressed genes from endometriosis patients, most were upregulated, including *APOE, DUSP1, G0S2, H2AFZ, ID4, MGST1* and *WEE1*. *PXK* was the only downregulated gene.	(1) Some endometriosis oocytes behaved similarly to healthy oocytes, need for increased sample size to further understand the transcriptome dynamics.	71
Zhao et al., 2019 [18]	Mechanisms coordinating oocyte maturation	qRT-PCR Single Cell	Sequencing (all)	Six human oocytes, including three IVM and three in vivo oocytes	Impairment of Krebs Cycle genes (*ACAT1* and *HADHA*) in IVM oocytes. Overexpression of *DPYD* (DNA Double strand break repair) in IVM, increased risk of double stand breaks or chromosome segregation.	(1) Sample size.	69
Qi et al., 2020 [19]	To understand differences in gene expression between healthy and PCOS oocytes	SMART-seq2 protocol Single cell	Sequencing (all)	14 oocytes (6:5:3) from 7 healthy women and 20 oocytes (7:4:9) from 9 PCOs patients (GV:MI:MII)	Downregulation of *CYP26A1* (meiosis-inhibiting), *MTRNR2L1*, *ELOA* and upregulation of *FAM53A* (ovarian carcinoma) in PCOs. Different developmental stages are relatively isolated at the transcriptomic level. Some mitochondrial functions may be prematurely activated at GV stage in PCOs.	(1) Sample size. (2) Need for independent validation wit qRT-PCR.	79
Barone et al., 2020 [20]	To understand the molecular basis of age-related chromosome mis segregation in human oocytes based on transcriptomic profiles	aCGH for aneuploidy; Ovation SoLo RNA-seq Single cell	Sequencing (all)	20 MII oocytes, divided into groups according to female age (<35 and ≥35). Corresponding CCs analysed.	No significant differences in gene expression in CCs according to age groups. Gene expression profile changes with age (1852 differentially expressed genes). *ESCO1, ESCO2, ESPL1, SMC1A* and *ATAG3* genes decreased (not statistically) in older group. *REEP4* downregulated in older patients.	(1) Sample size. (2) More variables related to female infertility ought to be studied. (3) Only MII stage included.	72
Yu et al., 2020 [21]	Gene regulation during oocyte maturation	Whole genome bisulfite sequencing (methylome) and SMART-seq (transcriptome)	Sequencing (all)	53 oocytes (17 MII, 17 MI, 19 GV).	Oocyte maturation stages (GV/MI vs MII) can be uniquely identified based on their transcriptome. *RBBP7* as being highly expressed in MII oocytes compared to MI. *PTTG1* and *TUBB8* were among the highest expressed genes in MII oocytes. Majority of differentially expressed genes were downregulated in MII. DNA methylation correlates with gene expression in oocytes.	(1) Heterogeneous group. (2) Sample size, 17 women, female [27-41 years of age].	92
Yuan et al., 2021 [22]	To investigate ageing-associated gene expression signatures of MII	scRNA-seq	Sequencing (all)	12 MII (n=6 ≥40y and n=6 <30). Three young eggs were excluded.	Differential gene expression was noted between age groups. *UBE2C* as a high-rank upregulated gene in older patients. Downregulation of genes involved in oxidative phosphorylation (i.e. *COX6C, CYTB, ND4L*)	Unclear if unfertilised oocytes had been ICSI’d before donating to research. Sample size. No comparison with GV stages.	71
Llonch et al., 2021 [23]	To study the effect of maternal age on the transcriptome of germinal vesicle stage (GV) and in vitro matured (IVM-MII) oocytes	scRNA-seq	Sequencing (all)	40 GV and 32 IVM-MII 37 patients (18-43y)	Over 1700 genes changed in transcript representation in correlation with women's age. *CPEB3* decreased with age. *BNC1* as a potential upstream regulator in in vitro matured. *PRDX1* and *GPX1* (antioxidants) downregulated with age. Upregulation of *ANAPC11, ANAPC15* and *AURKA* with increased BMI.	IVM cohort was cultured for 30 hours, may not be reflective of MII oocytes maturing at the optimal time.	92

## Results

**Fig. 1:**
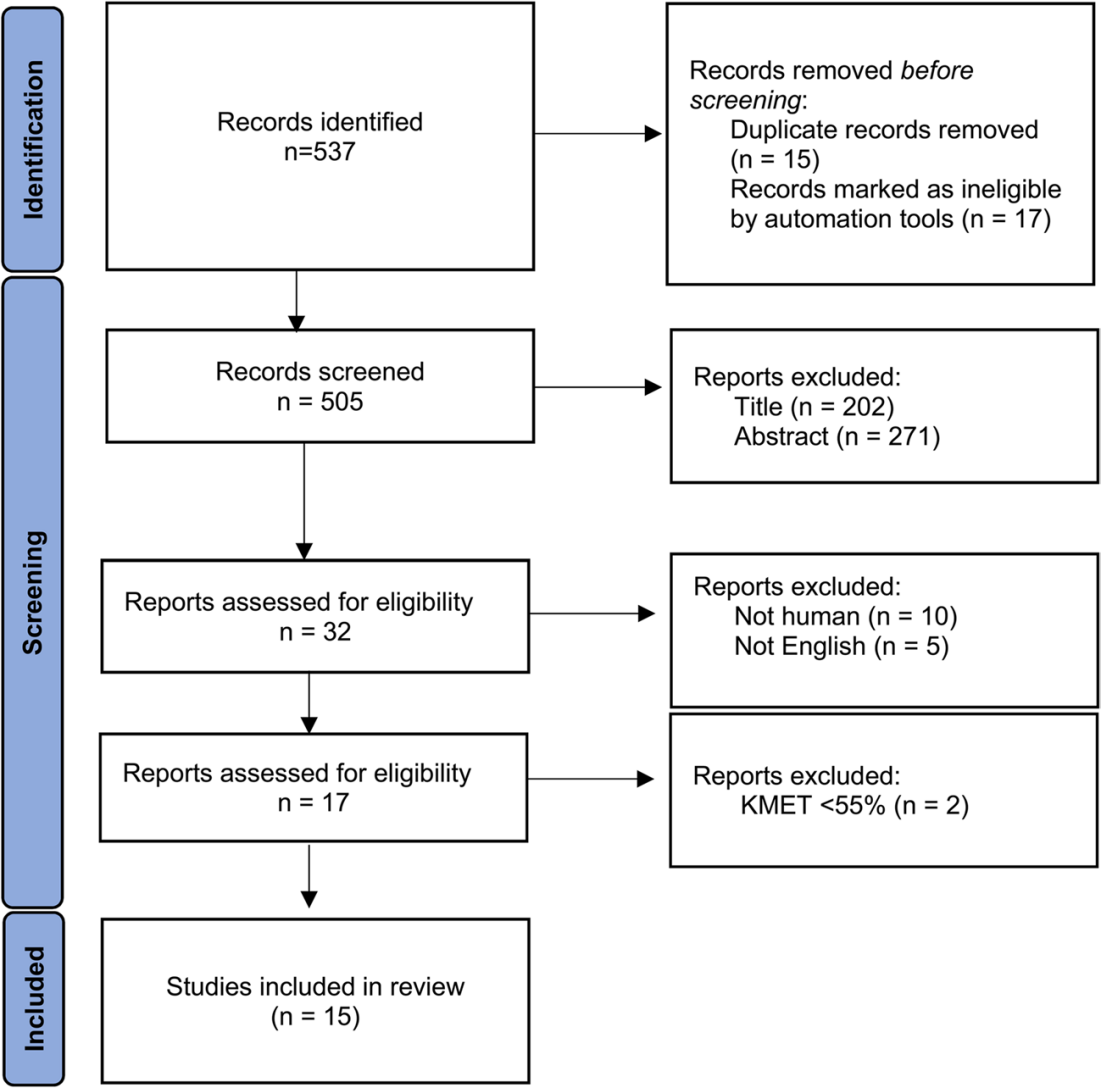
PRISMA Flowchart. Summarised detailed the database searches, the number of studies screened, and the full texts retrieved

The initial search from the three databases resulted in 505 records, once duplicates were removed. After screening by title and abstract, a total of thirty-two publications were subjected to the eligibility criteria and seventeen were further appraised using KMET quality score. Fifteen original articles were included for data extraction and analysis (Fig. 1).

### Study characteristics

Table 1 contains the synthesis for the included studies. A total of six studies were performed in China while other studies were included for each of the following countries: United States (*n*=2), Spain (*n*=3), Italy (*n*=2), and Iran (*n*=2). The majority (14/15, 93.3%) based their results on single cell approaches for human oocytes and only one study used pooled oocytes. Moreover, ten studies performed a comprehensive sequencing whereas a minority (5/15, 33.5%) looked into specific genes. The KMET score of the included quantitative studies ranged from 60 to 92%. The main limitation found were limited sample size, control for confounding variables and lack of report of estimate of variance for the main results. Oocyte maturation (including regulation of cell cycle progression and cellular metabolism) is the most studied theme, being included in twelve out of the fifteen selected studies. Genes involved in fertilisation, and embryo development were discussed in four and three research studies, respectively.

### Regulation of the cell cycle progression

Oocyte maturation is the culmination of an intermittent process which involves two arrests during the meiotic cycle (prophase I and metaphase II) together with changes at a cytoplasmic level. Resumption of the cell cycle is orchestrated by cytoplasmic factors, but its progress depends on checks for adequate environmental conditions, DNA integrity, DNA replication completeness and chromosome spindle attachment [25]. High concentrations of cyclic adenosine monophosphate (cAMP) are imperative to keep the cycle arrested. Cyclin-dependant kinases (Cdk), together with cyclins, drive the cell cycle forward once cAMP levels decline [26]. The maturation-promoting factor (MPF) is a Cdk-cyclin complex that targets the nucleoplasm and is activated in nucleoplasm (such as by SGK1[27]) thus promoting chromosome condensation and nuclear envelope breakdown as an initial step in prophase I that precedes spindle formation and progression to metaphase I.

A comparison of expression profiles in 120 GV stage oocytes with and without cytoplasmic transfer (CT) from MII-stage oocytes at retrieval day, was performed to evaluate the cytoplasmic effect to promote maturation in immature eggs [10]. The authors showed that meiosis resumption involves not only specific gene expression but also has a time-dependant dimension as culture for 24h of GV oocytes improved *CDC25* and *AURKC* expression and meiosis progression compared Day 0 GVs (regardless of CT). Interestingly, expression of DNA repair transcripts (BRCA1, ATR and ATM) showed to be increased after 24h culture, suggesting that DNA repair was active at that stage, which was not observed in immature oocytes at the day of retrieval. DNA repair, rather than apoptosis, was suggested as inhibitors of apoptosis such as neural apoptosis protein (NAIP) exhibited increased expression [10]. Related to nuclear maturation, research has shown that AURK transcripts are overexpressed in women with increased body mass index, which could explain described spindle abnormalities in oocytes (as shown by Machtinger et al., 2012) [23, 28].

Additionally, overexpression of DPYD (a NADP+ -dependent enzyme encoded with a role in the repair of DNA double strand breaks) has been reported in in vitro (IVM) studies, showing that oocytes have compensatory mechanisms to minimise maturation failure [18]. A study of nine human MII showed that, the biological processes that showed the most differential enrichment between old and young oocytes were those related to ubiquitination and the ubiquitination-related pathway, including the mitotic cell cycle and meiosis [22]. The overexpression in older patients of key transcripts such as CDC34, UBA1, and UBE2C, as well as the under expression of the hub gene *SKP1*, are associated with an enrichment in the “Ubiquitin-mediated proteolysis” pathway. The authors explain that elevated levels of UBE2C result in the premature activation of the APC and cytokinesis, hence disrupting the meiotic cycle.

MAPK activity could be inhibited in presence of oxidative stress, and DUSP1 overexpression could play a part in this cause-effect phenomenon as described in woman with endometriosis [17]. In the same study, looking at 32 MII-stage oocytes from healthy versus woman with endometriosis, it was found that WEE1 was upregulated in the latter group. Together with *DUSP1* and *WEE1*, other genes such as *ID4*, *G0S2*, and *CYP26A1* could be useful as markers to understand meiotic maturation inhibition rates in oocytes [17, 19]. Using a single-cell transcriptomic approach, obesity resulted in differential gene expression along different maturation stages [15]. *DUSP1* was also shown to be upregulated together with the inflammatory gene *CXCL2* whereas *TWIST1*, *ID3*, *GAS*, and *TXNIP* were downregulated. Ovarian proinflammatory signalling is poorly understood in the context of oocyte maturation.

Relevant to meiosis II arrest maintenance via protein kinase C (PKC) inhibition, *ANXA5* over expression in 36 vitrified/warmed mature oocytes has been hypothesised to contribute in younger females with good ovarian follicle count (AFC), which could be a favourable marker linked with prevention of premature granule exocytosis [13]. Low AFC, associated with increased risk of infertility, showed an increased expression of a microtubule-severing enzyme (Fidgetin, FIGN), which could represent a marker of developmental quality in oocytes and could be related to oocyte aneuploidy. Analysis of genes associated to age and genome integrity on 20 MII-stage oocytes showed a non-significant decreasing trend of expression of genes belonging to the cohesin pathway (*ESCO1, ESCO2, ESPL1, MAU2, SMC1A, SMC1B*, *and STAG3*) in the ≥35 years age group [20]. In the same research, it was found that *REEP4* (encoding for a microtubule-binding protein) is down-regulated in elder patients, which could induce chromosome mis segregation and perturbations of nuclear envelope.

An interesting gene whose expression may respond to metabolic changes and induce spindle abnormalities is ECAT1, coding for a subunit of the subcortical maternal complex [29]. It has been predicted to be a maternal gene as its mRNA is highly expressed at the GV stage and decreases throughout oocyte maturation, being almost absent in pre-implantation embryos [11]. Its function as a regulator of the cell cycle has been hypothesised since its downregulation using short interfering RNAs resulted in less oocytes undergoing germinal vesicle break down (GVBD). Moreover, it is thought that ECAT1 could impair SAC function as knockdown model results in higher rates (88%) of abnormal spindles in MII- stage oocytes compared to the control group (33%). Securin and beta-tubulin subunit proteins encoding genes (*PTTG1* and *TUBB8*) were highly expressed in mature oocytes when compared to immature stages, which are relevant to chromosome segregation and maturation progression [21].

Cytoplasmic polyadenylation plays a critical role in controlling the stability and translation of maternal-effect mRNAs during oogenesis. Research has shown that a decrease in CPEB2 mRNA levels with aging may be associated with the diminished quality of mature MII oocytes that were subjected to in vitro maturation, leading to impaired protein production [23]. The authors also identified the zinc finger transcription factor BNC1 as a potential upstream regulator in in vitro matured (IVM) MII-stage oocytes, which could establish a relationship between deficient nuclear maturation and ageing. In the same study, *SON* was identified as another potential master regulator. *SON* is responsible for encoding an RNA-binding protein that promotes pre-mRNA splicing, especially in transcripts with weak splice sites and those involved in cell cycle and DNA-related processes [23].

### Cellular metabolism

**Fig. 2:**
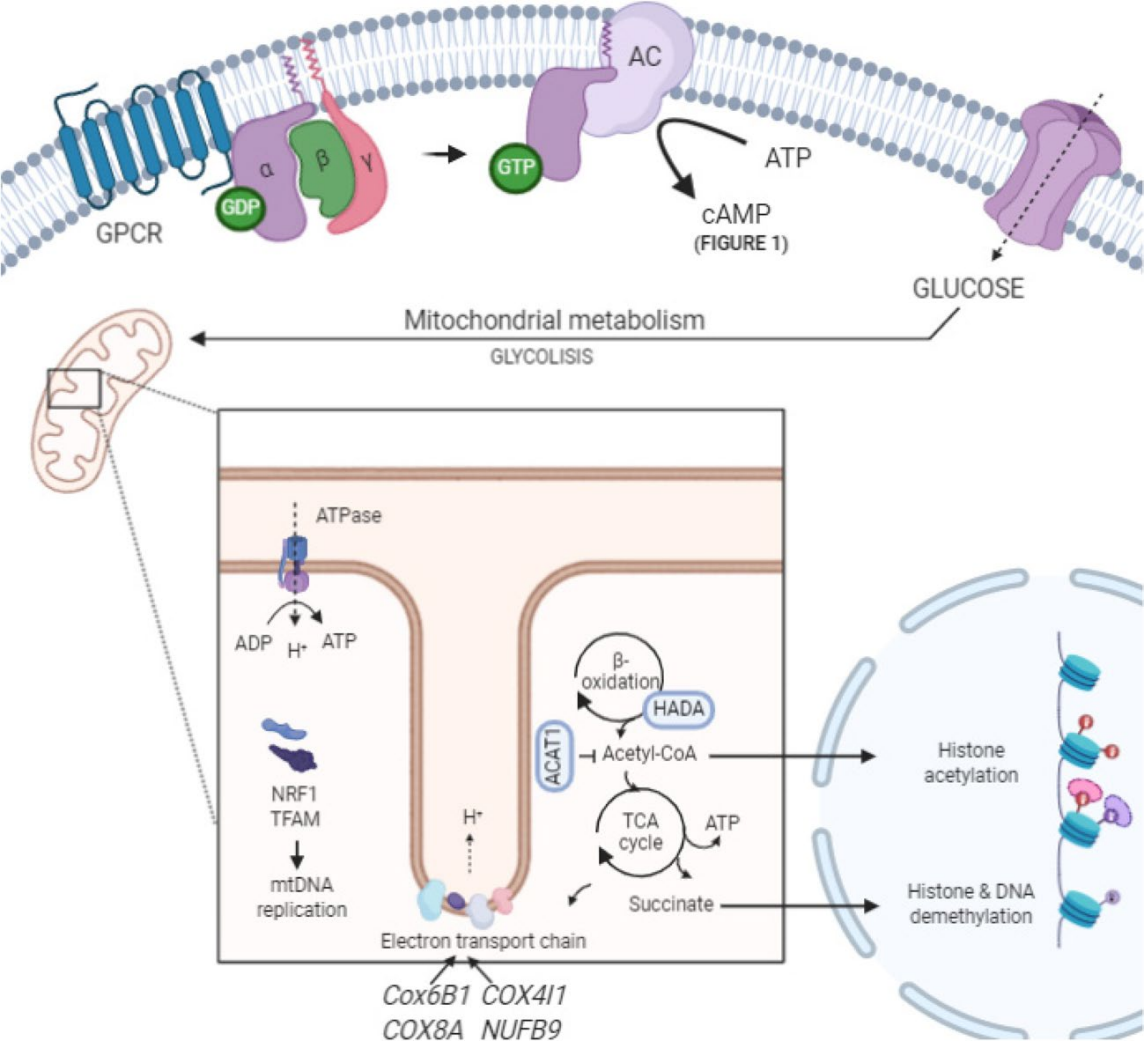
Cellular metabolism interactions. Many cellular processes need energy for their correct functioning, including spindle assembly and stability. Some genes have been shown to orchestrate mitochondria copy number replication, indispensable for homeostasis maintenance; whereas others play a role in β-oxidation and TCA cycle which could have a direct effect in DNA acetylation/ demethylation. *Original diagram*, Created with BioRender.com

Like any other cell type, oocytes require complex sequences of controlled biochemical reactions in order to sustain life and viability. An increasing research in metabolomics has been noted in the IVF field in order to identify and quantify intracellular and extracellular metabolites to, in turn, define the environment and understand its relationship with oocyte competence. Mitochondria are the major site for energy generation (ATP) in cells since a large number of enzymes involved in different metabolic pathways are contained within their matrix (Fig. 2). The cumulus cells in presence of functional gap-junction communication support the oocyte with sufficient amounts of pyruvate, lactate, and nicotinamide adenine dinucleotide phosphate (NADPH) [30].

Related to the function and mitochondrial performance in human oocytes, comparison of 10 GV, 9 MI and 8 MII-stage oocytes revealed increasing levels of *TFAM*, *NRF1*, and *MT-CO1* gene expression along maturation stages [9]. *NRF1* and *TFAM* genes regulate mitochondrial DNA (mtDNA) copy number, thus providing an adequate energetic pool in oocytes required for homeostasis maintenance and regulation of survival. Also related to mitochondrial function, *ATPase6* gene has been suggested as a marker of maturation since its transcript level is higher in IVM compared to immature oocytes [10]. It is noteworthy to highlight that some metabolic pathways and related genes have been shown to be significantly impaired in IVM oocytes [18]. Using qPCR, authors found that in IVM oocytes compared with the control group, expression of *ACAT1* and *HADHA* was considerably reduced, whereas DPYD expression was high. *ACAT1* and *HADHA* genes shared eight metabolic pathways and their deficient expression resulted in acetyl-CoA and succinate production blockage, resulting in declining the energy metabolism. The same study showed that ATP content of IVM oocytes was significantly reduced with the aid of immunofluorescence. This aberrant metabolic environment was also suggested to not only impair ATP production but also calcium signalling. Interestingly, authors showed a compensatory mechanism based on nicotinamide nucleotide transhydrogenase (NNT) transcript level, where NADP+ and NADH are generated – although this does not correspond to the predicted transcriptional silencing during maturation.

Mitochondria-related genes such as *COX6B1, COX8A, COX4l1*, and *NDUFB9* have been shown to be highly expressed in healthy MII-stage oocytes compared to immature oocytes [19]. Remarkably, authors showed that in patients with polycystic ovary syndrome, immature oocytes highly expressed those genes suggesting a premature activation of oxidative phosphorylation process in mitochondrial function. The aberrant status of mitochondrial energy metabolism in this scenario highlighted the connection between metabolism and oocyte quality. Other research has shown downregulated expression of genes involved in oxidative phosphorylation pathways in the context of oocyte ageing [22]. Cell-surface receptors such as G-protein-coupled receptors (GPCRs) play a pivotal role in metabolic processes as they catalyse the synthesis of cAMP from molecules of ATP. These GPCRs have been shown to be age-sensitive in human oocytes [20]. Dysregulated expression could lead into premature meiotic resumption, thus compromising oocyte quality as showed in other animal models [31].

Commonly stored in the form of droplets, lipids provide a vast potential energy reserve in oocytes. The increased gene expression of apolipoprotein E (*APOE*), involved in the metabolism of lipids, has been noted in patients with endometriosis compared to healthy woman. Given its role in lipoprotein metabolism and lipid transport, *APOE* overexpression could translate in increased lipid metabolism resulting in increased oxidative stress [17]. In the same cohort, authors described upregulation of *G0S2* (mitochondrial protein which promotes apoptosis via BCL2) and *ID4* (related to decreased cell proliferation). In a different study, two of the transcripts that showed a decline in representation in GV oocytes with advancing age were GPX1 and PRDX1, which play a vital role in safeguarding cells against oxidative damage [23].

### Fertilisation

For the female and male gametes to interact, the sperm needs to overcome the different barriers that the oocyte presents, namely the cumulus cells and the zona pellucida (ZP) [32]. The zona pellucida is a glycoprotein matrix synthesised by the oocyte and surrounds it. ZP3 glycoprotein interacts with receptors in the sperm membrane which will lead to the fusion of its membrane to the oolema. Fusion of membranes will trigger molecular changes using calcium as a second messenger, resulting in meiosis II resumption and fusion of pronuclei. mRNA expression of ZP genes (*ZP1-4*) in 98 oocytes at different maturation stages, showed a significant decrease in ZP 1, 2 and 4 together with a non-significant decrease in *ZP3* expression from GV to MII-stages [14].

Female age has shown a differential transcriptomes in human oocytes [13]. Increased in expression for the younger groups, *ANXA5* codes for a protein which interacts with protein kinase C, a key regulator of fertilisation events. Increased expression of *PRRG1*, a calcium ion binding protein gene, was found in older women which could impair fertilisation events. Similarly, gene expression alterations induced after cytoplasmic transfer from a mature to an immature oocyte could lead to calcium impairment, which could lead to fertilisation failures [10]. Because of its involvement in the subcortical maternal complex, *ECAT1* expression levels could ascertain the fertilisation potential in oocytes, as correct pronuclear formation has been seen to decrease from 84 to 54% when *ECAT1* gene was silenced [12].

### Embryo development

Oocyte-to-embryo transition is nature’s masterpiece involving syngamy of the gametes, genetic combination, orchestrated synchrony of divisions, cell fusion, cellular differentiation, expansion and hatching which culminates in embryonic genome activation (EGA). The key regulators coordinating this transition and EGA in human continue to be weakly understood [33]. Upon fertilisation, the zygote will divide to form an embryo and by day 3 of embryo development, human embryos ought to have six to eight cells. Because of its role in maintaining the accuracy of spindle assembly, impairment of genes such *ECAT1* result in reduced cleavage rates (abnormal divisions) [11]. Hence *ECAT1* disruption was not only associated with compromised maturation but also with embryos which fail to develop. Interestingly, analysis of 39 oocytes from 24 women showed that body composition may influence mRNA stability and that obesity leads to an upregulation of proinflammatory-related transcripts in oocytes, which in turn result in altered gamete and embryo development [15].

A study analysing oocytes, cleavage embryos and blastocysts revealed that for some proteins which are initially maternally derived, mRNA expression could sustain their concentration past EGA [12]. Immunofluorescence for PLAC8 protein showed its presence at beyond the morula stage, although PLAC8 mRNA expression was not detected in MII oocytes or cleavage stage embryos (PLAX8 protein was detected in MII). This protein has been appointed as a potential biomarker of implantation potential as negative outcomes are associated with no protein detection [12]. PLAC8 protein could be related to expansion process once embryos implant.

## Discussion

The present review highpoints the degree of orchestration of different processes, such as cell cycle progression, cellular metabolism and activation for zygotic/embryonic transcription, in order to establish human oocyte quality.

Looking at oocyte transcriptome, it has been established that its maturation stage was the main factor defining internal composition with almost 6,000 genes differentially expressed between GV and MII stages [21]. The same group identified around 450 genes uniquely expressed in single stages. Although the presence of some transcripts may naturally happen due to progression and arrest of the cell cycle, others have been shown to be susceptible to external factors such as female age, obesity and or medical conditions (i.e. endometriosis). This draws scientists to question how much of oocyte quality is established from start (“nature”) and how much relates to cell plasticity changes in response to external factors during development (“nurture”) as it has been shown that mature oocytes have been found to be surrounded by cumulus cells with distinct gene expression profiles [14].

As female age increases, the percentage of poor-quality oocytes also increases, starting at 50% at 20 years of age and reaching 95% at 35 years of age [34]. Literature reviewed in this article has illustrates the relationship between patient demographics and chromosome missegregation, perturbations of nuclear envelope and premature maturation. Furthermore, alterations in metabolic pathways have been described to affect maturation and genetic makeup of oocytes [35, 36]. The present work has reviewed literature showing how some genes related to mitochondrial activity show upregulation in mature stages, compared to immature oocytes. Nevertheless, in vitro maturation of non-competent oocytes may not be a viable option at present as such matured eggs showed some degree of mitochondrial function impairment. Recent mouse model research suggests that supplementing growth hormone during in vitro maturation (IVM) of oocytes could be a potential strategy to improve the success rate of ART by reducing cAMP levels, promoting mitochondrial function, reducing DNA damage and apoptosis [37]. Recent literature has provided improved IVM protocols such as two phase IVM with pre-maturation steps, e.g. initial GV arrest followed by in vitro maturation using EGF-like growth factors or CNP peptide [38–40]. Such systems, have reported a significantly improved maturation and clinical pregnancy rates versus standard IVM in certain patient cohorts.

Cytoplasmic transfer (CT), from a mature oocyte to an oocyte with cytoplasmic alterations, has been proposed as a technique to restore normal growth and function. Although further evidence is still required, this may only be relevant when performed between two mature oocytes. Hoseini et al., 2016 suggested that such technique was not effective to restore cytoplasmic maturity of recipient GV oocytes [10].

Interestingly, some genes (i.e. *ECAT1*) found in oocytes were identified to be relevant not only during maturation but also during embryo development. Moreover, literature has illustrated how some maternally inherited proteins (i.e. PLAC8) are dynamic and expression may be found across oocyte and embryo development. Such studies have described how protein expression may not be necessarily related to mRNA expression. Although, measure of RNA levels up to day 3 of embryo development is not direct proof of function as this RNA is *silent*. Recently, new methodologies have combined transcriptome and translatome sequencing to study gene expression in oocytes. Research has found that translatome could be more accurate measure of gene expression than transcriptome, especially in cells that undergo translational regulation like oocytes [41].
